# Annotations

**Published:** 1894-12-22

**Authors:** 


### Annotations.

### Diphtheria: "the Brute Force of Figures."

Professor Virchow considers it to be "the duty of every practitioner to use antitoxic serum in the treatment of diphtheria." This is the weighty dictum of a serious man and compels consideration. Why has Virchow come to this conclusion? Not because laboratory experiments have shown certain effects upon animals, true and important though this be, but because of what he calls "the brute force of the figures" which have been accumulated in the Kaiser and Kaiserin Friedrich Hospital. A brief summary of these figures and facts will demonstrate their marked significance. During a period of twenty-one weeks all the cases of diphtheria were treated as experimental cases. In the first eight weeks the serum alone was used upon all the patients. At the end of the eight weeks the supply of serum fell short. Thereupon for seven full weeks the ordinary methods of treating diphtheria were fallen back upon. At the end of these seven weeks, the results not being so good as for the previous eight weeks, the serum treatment was resumed for a period of six weeks. Here are the actual figures for the three series of weeks, and whoever ponders them will conclude that Virchow employed a metaphor by no means too strong when he spoke of the conviction brought to his mind by their "brute force." In the first eight weeks, the serum being the only treatment, 62 cases were treated, of which 55 were cured and 7 died. In the next seven weeks, the serum having given out and old-fashioned methods being resorted to, 110 cases were treated, of which 54 were cured and 55 died. In the final series of six weeks 81 cases were treated, and this time by the serum again. Of these 69 were cured and only 12 died. In other words, the serum treatment up to the present time has given a mortality of only 13·2 per cent. of deaths, whilst the older methods in use at Berlin have shown a mortality of 47·8, or almost a half. Such are Virchow's data. "All theoretical considerations," says this eminent and venerable scientist, "must give way before the brute force of these figures." Most readers, whether medical or non-medical, will, we think, be inclined to agree with him.

### The Doctors and the Midwives.

Quite a bombshell has been dropped into polite medical circles by the action of the General Medical Council, in declaring that the issue of the midwifery certificates of the Obstetrical Society of London, in their present form, will in future be regarded as "conduct infamous in a professional respect." Short of removal from the register this is the severest punishment which the council can apply, and it is a matter for great regret among its friends, and for unholy laughter among its enemies, that an official body like the General Medical Council should have chosen first of all to discuss a public matter like this *in camera*, and then to do exactly what it has done. Nothing could detract more from its authority, and from its influence for good over those men who are wavering on the verge of evil courses than to see that its heaviest thunders are directed against a set of men who are everywhere respected, who are certainly the leaders in their department of medicine, and who have posed somewhat as the quintessence of professional propriety. More than ever is this action of the Medical Council to be regretted, inasmuch as the work of the Obstetrical Society in examining and certifying midwives has been a good work, and one of national importance. The evidence taken last year before the Select Committee on the examination and registration of midwives showed the injuries and the frightful mortality caused to women in childbirth all the country over by the ignorance of midwives, and, whatever the feeling of certain sections of the medical profession may be, the feeling of the public is in favour of the education and registration of these women; so much so, in fact, that this particular certificate has come to be publicly recognised as a diploma for its particular purpose, and the letters L.O.S. have come to be accepted, outside professional circles, as meaning a certified midwife. In this, no doubt, is the head and front of its offending; and in truth there is much to be said against private bodies giving certificates of this sort at all. But they fill a gap. The public demands the education and registration of midwives, and until that work is legally entrusted to some public body these L.O.S. certificates do at least ensure that their holders have received a training. It is all very well for the Medical Council to act as the cat's paw of the Lancashire and Cheshire Branch of the British Medical Association, but it will probably be found by this redoubtable branch that the stoppage of these voluntary certificates will be the very thing most likely to hasten on the legislation which they so much dread. The L.O.S. and other certificates at the least covered the ground, gave some protection, and offered a *modus vivendi*. If they are abolished the public demand for some means of distinguishing the trained from the absolutely ignorant midwife will all the more quickly eventuate in legislation, and in the establishment of a register under the sanction of the Government or the County Councils.

### Antitoxin in the London Hospitals.

At the meeting of the Clinical Society held on December 14th a paper was read by Drs. Washbourn, Goodall, and Card, giving the results of the treatment of diphtheria by antitoxin, which has been recently carried out at the Eastern Hospital, with serum supplied by the British Institute of Preventive Medicine. Of the 72 cases treated, which were "clinically" recognised as diphtheria, 14 died, which gives a mortality of 19·4 per cent. This is a considerable reduction as compared both with the previous mortality at the same institution and that current at the other hospitals of the Asylums Board. In fact, during 1893 the mortality at the Eastern Hospital of cases under 15 years of age (the limit in the cases treated) was 41·8 per cent., and in 1894, up to the commencement of the treatment, 36 per cent., giving an average of 38·8 per cent., exactly twice that which has occurred since the new treatment has been put in operation. It must be noted, however, that at the South-Eastern Hospital, during the time that the experiment was being carried out at the Eastern, the mortality was only 23·6 per cent., against an average for last year of 39·9 per per cent., showing how variable statistical results are apt to be when the numbers are small. We shall await with interest the result of the adjourned discussion at the Clinical Society.

